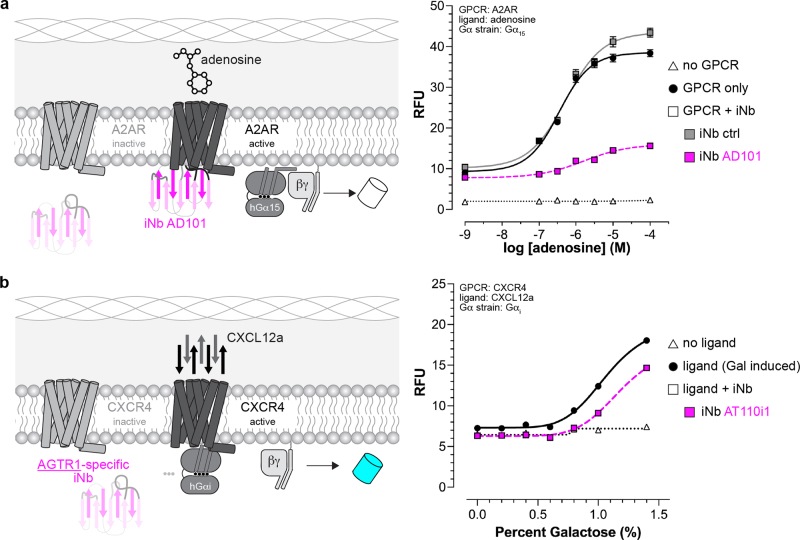# PIONEER: A periplasmic display platform for synthetic biology-based screening of genetically encoded protein regulators

**DOI:** 10.1016/j.jbc.2025.110967

**Published:** 2025-11-20

**Authors:** Jacob B. Rowe, Kyutae Lee, Daniel G. Isom

**Affiliations:** 1 Department of Molecular and Cellular Pharmacology, University of Miami Miller School of Medicine, Miami, Florida, USA; 2 Tumor Biology Program, University of Miami Sylvester Comprehensive Cancer Center, Miami, Florida, USA; 3 Frost Institute for Data Science and Computing, Miami, Florida, USA

**Keywords:** DCyFIR, GPCRs, genetically encoded modulators, nanobodies, SynBio, yeast

## Abstract

Periplasmic display in yeast is a promising but underdeveloped method for screening and studying genetically encoded biomolecules. We present Periplasmic dIsplay Of geNetically Encoded rEgulatoRs (PIONEER), a modular platform that localizes peptides, proteins, and nanobodies to the membrane-proximal periplasmic space of *Saccharomycescerevisiae*, enabling direct interrogation of membrane protein function. By optimizing secretion signals (ss) and display scaffolds, PIONEER ensures stable ligand retention and robust autocrine signaling. The ability to assess peptide and protein ligand activity through signaling, rather than binding alone, marks a key advance toward function-first screening. Applied to human G protein-coupled receptors, the system enables detection of surface expression, ligand activity profiling, and classification of nanobody regulators, including antagonists and conformational stabilizers. It also distinguishes intrabodies based on their functional effects and chaperone activity. Although demonstrated with G protein-coupled receptors, PIONEER is adaptable to a wide range of membrane and soluble protein targets. As AI-driven design expands the space of candidate ligands and binders, this platform offers a scalable method for linking predicted sequences and their structures to biological function.

Yeast is a versatile eukaryotic platform widely used in synthetic biology for applications ranging from the development and production of biopharmaceuticals (1), biofuels (2), sustainable materials (3), and natural products (4, 5, 6). For example, yeast has been engineered to synthesize human insulin (7), the anti-malarial drug artemisinin (8), and complex proteins requiring post-translational modifications like glycosylation and disulfide bond formation (1). Yeast systems are also used to design genetic circuits, enabling precise control of gene expression (9), and as biosensors for environmental or medical diagnostics (10). Furthermore, their industrial scalability makes them invaluable for large-scale production processes (11).

Yeast display is a foundational synthetic biotechnology that uses engineered yeast, typically *Saccharomyces cerevisiae*, to present proteins and peptides on the cell surface. The protein of interest is genetically fused to Aga2p, which is secreted and forms disulfide bonds with the cell wall-anchored Aga1p, tethering the target protein to the cell exterior (12). This configuration makes displayed proteins accessible for binding assays, high-throughput screening, and interaction analysis. As such, this technique is widely used for antibody affinity maturation through directed evolution (13, 14), nanobody discovery (15, 16), enzyme engineering (17), studying protein-protein interactions (18), and biosensor design (19).

In contrast, yeast periplasmic display is conceptually the inverse of surface display and remains vastly underexplored. It offers unique advantages, particularly for applications involving transmembrane proteins. This method localizes recombinant proteins to the space between the plasma membrane and the inner cell wall, a confined periplasmic-like compartment. Proteins fused to anchors such as invertase (Suc2p) or engineered Flo1p variants are secreted from the cytoplasm but retained near the membrane (20, 21, 22), enabling direct interaction with membrane-embedded targets.

Unlike surface display, which primarily supports the identification of binding partners, periplasmic display enables functional selection (Fig. S1). This allows for the discovery of genetically encoded peptides and proteins that not only bind but also modulate transmembrane protein activity through agonism, inhibition, or allosteric regulation. Because each ligand is barcoded in the yeast genome or on a plasmid, there is a one-to-one correspondence between function and sequence, simplifying downstream screening applications and identification.

Here, we present Periplasmic dIsplay Of geNetically Encoded rEgulatoRs (PIONEER), an advanced platform for discovering and characterizing genetically encoded modulators of G protein-coupled receptor (GPCR) signaling. Built on our previously established DCyFIR platform (21, 23, 24, 25), PIONEER employs a humanized yeast mating pathway along with optimized secretion signals (ss) and cell wall display proteins to support studies of peptide- and protein-based ligands. We demonstrate its utility for detecting GPCR surface expression, profiling ligand-activated signaling, and characterizing GPCR-targeting nanobodies. PIONEER provides a powerful tool to accelerate the development of next-generation GPCR modulators for structure-based drug discovery, basic research, therapeutic development, and theragnostic applications.

## Results

**Figure 1:**
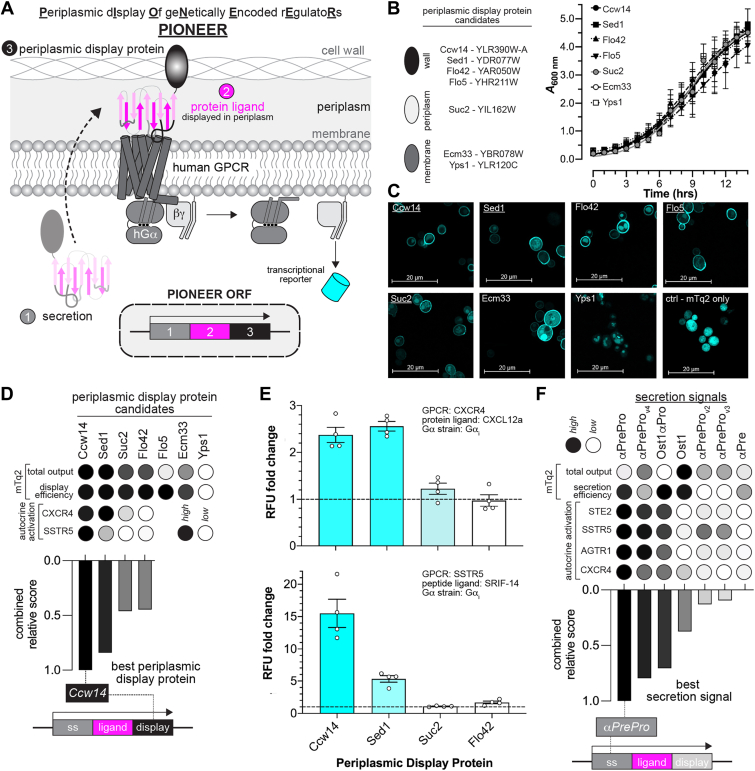
**Design and validation of the PIONEER platform.***A*, Schematic of the PIONEER system. Human GPCR signaling in the DCyFIR system is modulated by periplasmically displayed proteins and detected *via* transcriptional activation of a fluorescent reporter. *B,* effects of display protein overexpression on yeast growth, assessed by optical density at 600 nm (A_600_). All constructs included the αPre secretion signal for comparison. *C,* confocal microscopy of mTq2-tagged display proteins. *Underlining* indicates proteins with prominent peripheral localization. *D,* performance scoring of display proteins in mTq2-tagging and ligand-display assays. *E,* GPCR activation in response to periplasmically displayed ligands in the DCyFIR system, measured in relative fluorescence units (RFU). Fold change is calculated relative to signaling elicited by secreted-only ligands. *F,* performance scoring of secretion signals in mTq2-tagging and ligand-secretion assays. Data in *B–F* represent mean ± s.d. of *n* = 4 or 8 biological replicates (as indicated for each panel). Ligand-based assays (*D–F*) were performed in a DCyFIR G_αi_ strain, except for *STE2*, which was tested in the parental yeast strain expressing native G_α_ (*Gpa1*). Rankings were normalized to the best- and worst-performing candidates in each assay. GPCR, G protein-coupled receptor; RFU, relative fluorescence units.

To optimize periplasmic display, we developed a modular platform in *S. cerevisiae*, using humanized GPCR signaling as a proof of concept. As shown in Figure 1*A*, the platform comprises three key components. First, an efficient ss directs proteins to the periplasmic space. Second, a genetically encoded protein ligand serves as the functional input. Third, a cell wall display domain retains the ligand in the periplasm, presents it on the inner cell wall, and preserves access to membrane-localized receptors.

While our demonstration centers on GPCR signaling and builds on the DCyFIR platform, we aimed to design a ss and display domain applicable across synthetic biology. Flo42, the current standard for periplasmic display, is a truncated, glycosylphosphatidylinositol (GPI)-anchored form of the yeast Flo1 protein but yields weak receptor activation compared to exogenous ligand addition (21). This limited performance may stem from low ligand-Flo42 expression or secretion, or structural interference in the fusion constructs. To improve display system performance, we focused on identifying and validating next-generation display domains.

## Ccw14, Sed1, Suc2, and Flo5 are effective periplasmic display scaffolds

**Figure S2:**
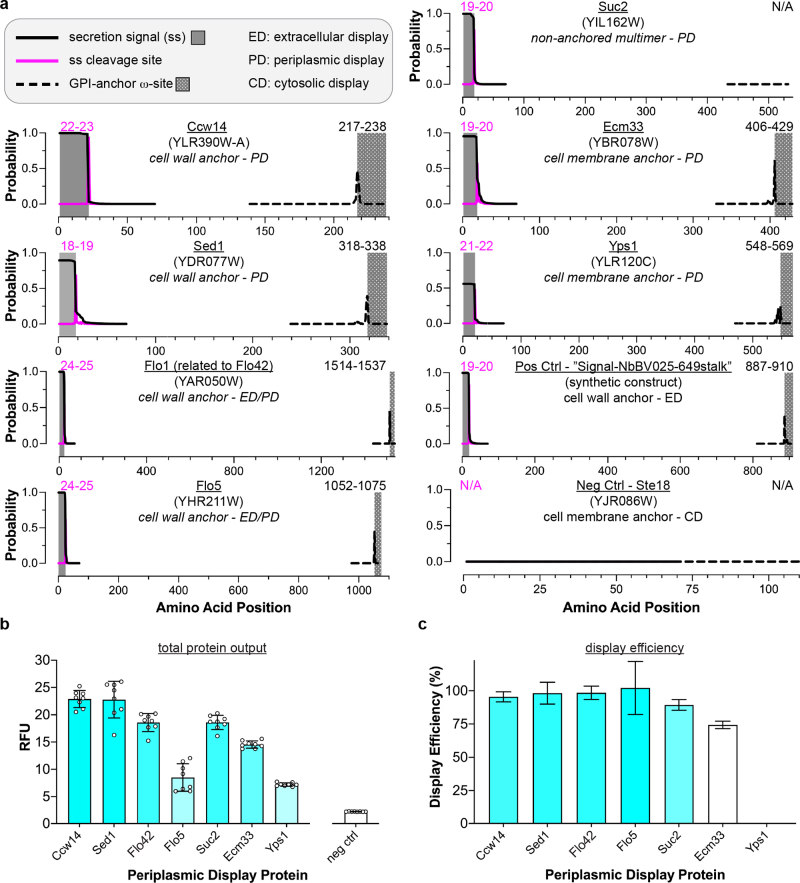


We began by surveying the literature and selecting seven periplasmic display proteins for testing based on relevance and feasibility (Fig. S2*A*). This set comprised proteins that reversibly bind the cell wall (Ccw14, Sed1, Flo5), diffuse within the periplasm (Suc2), or anchor to the cell membrane (Ecm33, Yps1). We included Flo42 as a benchmark. As shown in Figure 1*B*, all yeast strains overexpressing a candidate reached stationary phase within 12 h, with final optical densities at 600 nm ranging from 3.5 to 4.5. These results indicated that overexpression did not impair growth or cause detectable toxicity.

Next, we examined the localization of each display candidate using mTurquoise2 (mTq2), a bright cyan fluorescent protein, as a test cargo. To ensure consistent targeting to the secretory pathway, we replaced each candidate’s native ss with αPre, a 19-amino acid signal derived from the yeast α-factor (26), a secreted peptide hormone that triggers mating responses. This yielded a set of αPre-mTq2-*X* fusion constructs, where *X* was the display candidate. We cloned each construct into expression plasmids, introduced them into yeast, and visualized localization using confocal microscopy.

As shown in Figure 1*C*, Ccw14, Sed1, Suc2, and Flo5 formed distinct mTq2 fluorescent rings around each yeast cell, with minimal signal in the cytoplasm and organelles. This pattern indicated efficient entry into and transit through the endoplasmic reticulum and Golgi. In contrast, Flo42, Ecm33, and Yps1 showed significant mTq2 fluorescence in vacuoles, suggesting misrouting and degradation. This likely explains the weak signaling observed in our prior studies using Flo42-trapped GPCR agonists (21). As expected, non-secreted mTq2 accumulated in the cytoplasm and produced bright, diffuse fluorescence. Notably, the fluorescence intensity in Figure 1*C* does not reflect total mTq2 levels, as laser power was adjusted to optimize image clarity. Based on localization patterns, we advanced Ccw14, Sed1, Suc2, and Flo5 for further testing.

## Periplasmic display domains effectively prevent detectable cargo escape

Next, we quantified the cell wall display efficiency of our top candidates using disqualified constructs as controls. First, we measured total mTq2 levels in yeast cultures (Fig. S2*B*). After pelleting, washing, and resuspending the cells, we measured retained mTq2 and calculated display efficiency as the percentage retained (Equation 1; Fig. S2*C*). As shown in Figure 1*D*, Yps1’s trapping efficiency was set to zero since it was not displayed (Fig. 1*C*). Flo5, despite its promising localization pattern, showed mTq2 output as low as Yps1 and was disqualified (Fig. S2*B*). All other candidates, including the controls, exhibited high display efficiency, indicating full mTq2 retention. These results demonstrate that our display domains effectively prevent detectable cargo escape.

## Ccw14 is the most effective scaffold for periplasmic ligand display

Compared to yeast surface display, periplasmic display remains underexplored in synthetic biology, with few studies exploiting protein retention between the plasma membrane and cell wall. Successful signaling applications require constructs that fold into active conformations, minimize steric hindrance of ligand-target interactions, and remain accessible to the extracellular face of transmembrane proteins. To define the scope and power of this approach, we used DCyFIR to screen synthetic autocrine agonism of human somatostatin receptor 5 (SSTR5) and chemokine receptor 4 (CXCR4) with Ccw14, Sed1, and Suc2 ligand-display fusions. Our results establish periplasmic display as a versatile platform for synthetic signaling, while revealing key parameters that govern ligand compatibility and scaffold selection.

**Figure S3:**
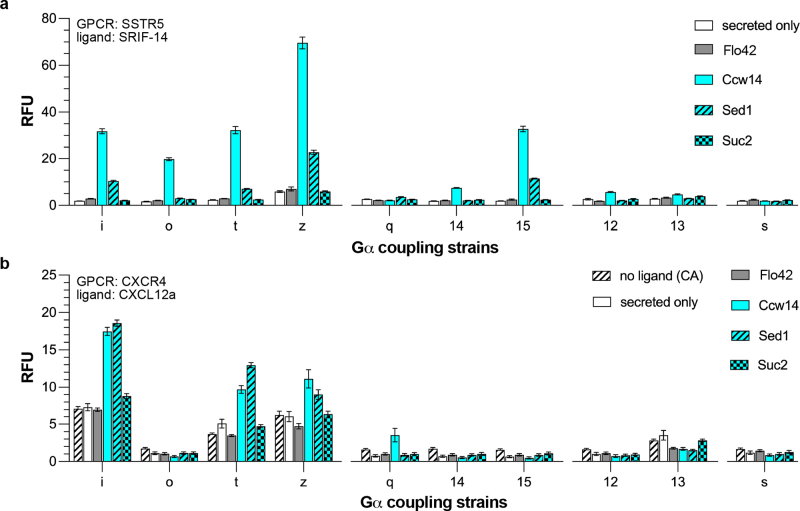


As shown in Figures 1, *D* and *E* and S3, genetically encoded αPre-display fusions enabled autocrine activation of CXCR4 and SSTR5, which recognize the protein ligand CXCL12a and peptide ligand SRIF-14, respectively. Ccw14 fusions performed best, driving strong activation for both receptors and yielding the highest combined relative score, reflecting total mTq2 output and signaling strength. Sed1 matched Ccw14 for CXCR4 but reached only ∼30% of its activity for SSTR5. Suc2 performed poorly, with activation comparable to the Flo42 control and less than half of Ccw14. These results established Ccw14 as the most effective scaffold for periplasmic ligand display.

For CXCR4 and SSTR5, displayed ligands engage the orthosteric site through N-terminal interactions, enabling compatibility with C-terminal Ccw14 fusions. In contrast, ligands that bind *via* their C-terminus may not be compatible with this design. Testing additional receptors showed that angiotensin receptor 1 (AGTR1) and the C5a receptor (C5aR) fail to activate when fused to Ccw14. Structural studies explain this incompatibility by showing that AGTR1, a key regulator of blood pressure and target of antihypertensive drugs, binds angiotensin II in a C-terminal-first orientation, with the C-terminal phenylalanine inserting deep into the orthosteric pocket to drive activation (27). Similarly, C5a engages C5aR by inserting its C-terminal tail into the transmembrane pocket, triggering conformational changes that couple G proteins (28). In both cases, a C-terminal Ccw14 fusion would occlude critical interactions and prevent activation.

## αPrePro is the optimal secretion signal peptide for Ccw14 display

**Figure S4:**
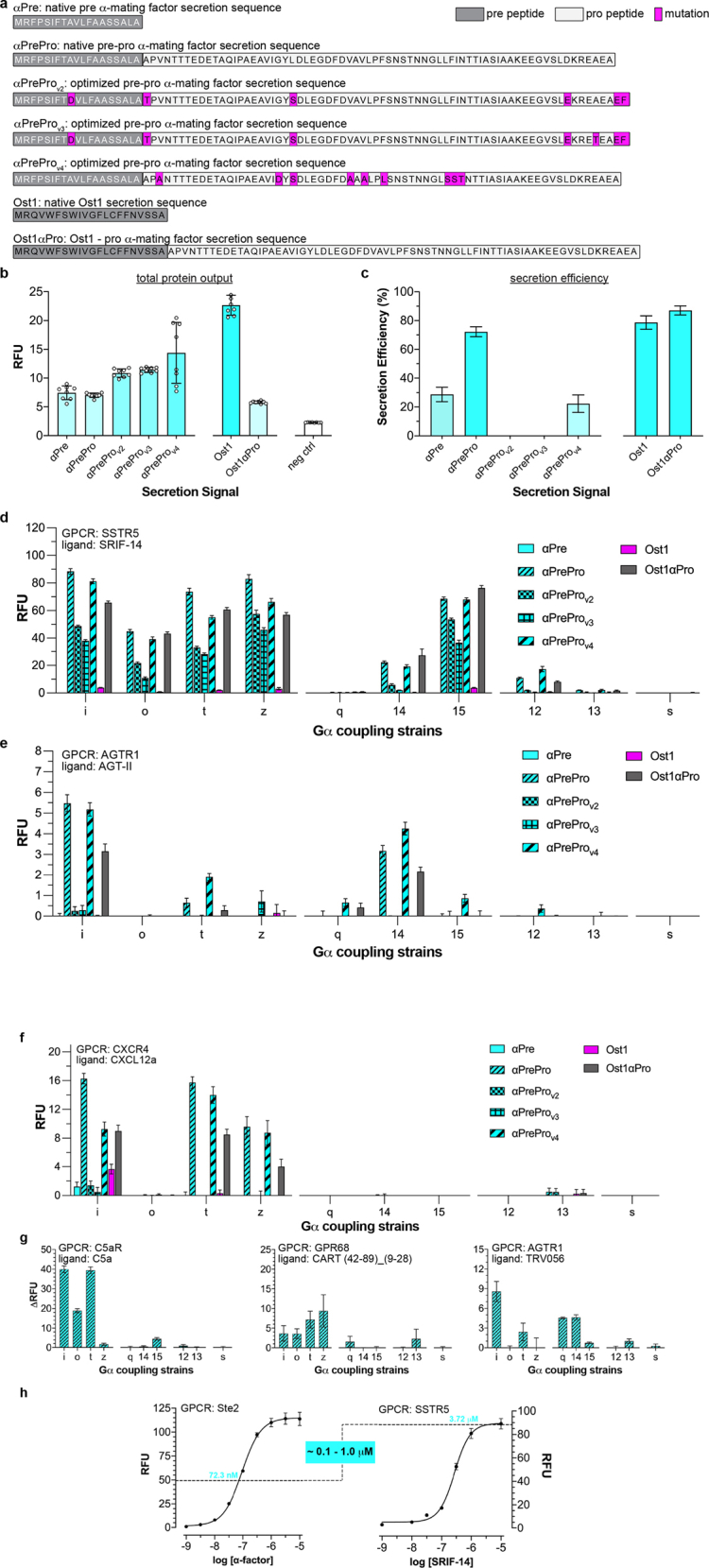


Having identified Ccw14 as the best display protein, we next optimized the signal peptide responsible for ligand secretion and signaling. Two ss’s from *S. cerevisiae* are commonly used in yeast biotechnology: αPrePro, which precedes the 13-amino-acid mating pheromone α-factor (26), and the 22-amino-acid N-terminal signal from Ost1, the α subunit of the oligosaccharyltransferase complex (29) (Fig. S4*A*). αPrePro is a bipartite signal comprising a 19-amino-acid αPre segment that directs posttranslational translocation into the ER and a 66-amino-acid Pro region that enables enzymatic processing and Golgi trafficking (30). In contrast, the Ost1 signal promotes cotranslational translocation into the rough ER and typically yields more efficient secretory entry (31). To improve displayed ligand secretion, we tested several αPrePro and Ost1 combinations.

As shown in Figures. 1*F* and S4, *B* and *C*, we evaluated seven *X*-mTq2 fusion constructs, where *X* denotes the ss’s. These included WT αPrePro and Ost1, the truncated αPre segment, three αPrePro variants (V2, V3, V4) with reported enhancements in Pro-region processing, and a hybrid Ost1αPro sequence previously shown to boost secretion in *Pichia pastoris* (30). For each construct, we measured total mTq2 output and secretion efficiency. Ost1 produced the highest output, followed by αPreProV4, V3, and V2, with αPre and αPrePro performing similarly. Ost1αPro yielded the lowest output. In contrast, secretion efficiency was highest for αPrePro, Ost1, and Ost1αPro, followed by αPre and αPreProV4, with V2 and V3 being the least efficient. Based on these results, we selected αPrePro, Ost1, and Ost1αPro as lead candidates.

We next evaluated which ss’s best supported autocrine GPCR activation in the DCyFIR system. Each secreted agonist functions as a pro-ligand that must undergo enzymatic maturation to remove its N-terminal ss’s and reveals the active form. To test this, we constructed a panel of *X*-agonist fusions, where *X* denotes the ss’s being tested. As shown in Figures 1*F* and S4, *D–F*, these included *X*-α-factor for Ste2, *X*-SRIF-14 for SSTR5, *X*-CXCL12a for CXCR4, and *X*-AGT-II for AGTR1. Among the ss’s tested, the native αPrePro consistently supported the strongest GPCR activation, followed by αPreProV4 and Ost1αPro. These results suggest that αPrePro undergoes more robust maturation at steady state, potentially due to parallel processing by dipeptidyl aminopeptidases such as Ste13 (26) and Kex2 (32), as well as the signal peptidase complex (33), which target conserved -X-Ala-motifs.

Considering total mTq2 output, secretion efficiency, and autocrine activation, we selected αPrePro as the optimal ss. To further validate its utility, we tested αPrePro-driven agonism and confirmed that it supported C5a activation of C5aR, CART peptide activation of GPR68, and TRV056 peptide activation of AGTR1 (Fig. S4*G*). To estimate the amount of autocrine ligand secretion, we next compared autocrine signaling outputs to dose-response curves generated with exogenously added peptide agonists. Specifically, we performed α-factor titrations of Ste2 and SRIF-14 titrations of SSTR5 and matched the efficacy and agonist concentrations between exogenous stimulation and autocrine activation. Based on this comparison, we estimated that the concentration of secreted autocrine ligand ranged between approximately 0.1 and 1 μM (Fig. S4*H*).

## PIONEERed luciferase complementation for detecting surface expression

**Figure 2:**
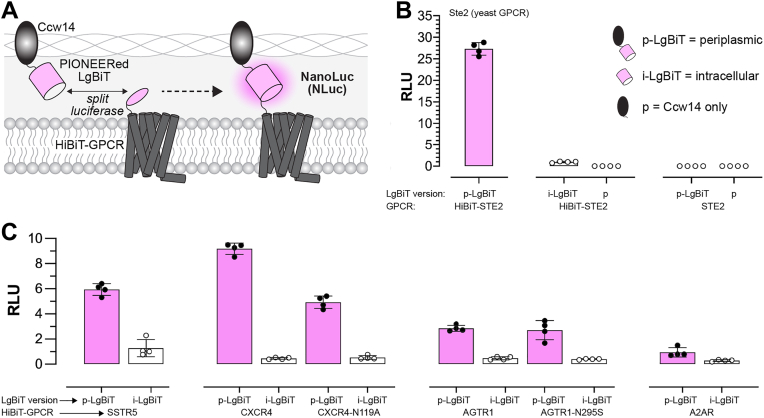
**Detection of GPCR surface expression using the PIONEER platform.***A,* schematic of surface detection using PIONEER. Periplasmically displayed LgBiT enables reconstitution of NanoLuc luciferase and luminescence upon interaction with GPCRs tagged at the N-terminus with HiBiT. *B,* surface detection of the native yeast GPCR Ste2 *via* luminescence reconstitution. *C*, Detection of human GPCRs expressed at the yeast surface using the same approach. Data in *B* and *C* are presented as mean ± s.d. of *n* = 4 biological replicates. Luminescence is reported in relative luminescence units (RLU). GPCR, G protein-coupled receptor; RLU, relative luminescence units; PIONEER, periplasmic display of genetically encoded regulators.

Having established PIONEER, we next sought to demonstrate its utility across different applications. We began by adapting a split luciferase system to detect cell surface expression. This system uses two fragments: LgBiT, a ∼18 kDa inactive luciferase, and HiBiT, an 11-amino-acid peptide that binds LgBiT with high affinity to restore activity (34). As shown in Figure 2*A*, we tagged the extracellular N-termini of GPCRs with HiBiT and periplasmically displayed LgBiT using an αPrePro-LgBiT-Ccw14 construct. Surface-localized GPCRs were expected to bring the HiBiT tag into proximity with the Ccw14-displayed LgBiT, reconstituting luciferase activity and producing a detectable signal.

**Figure S5:**
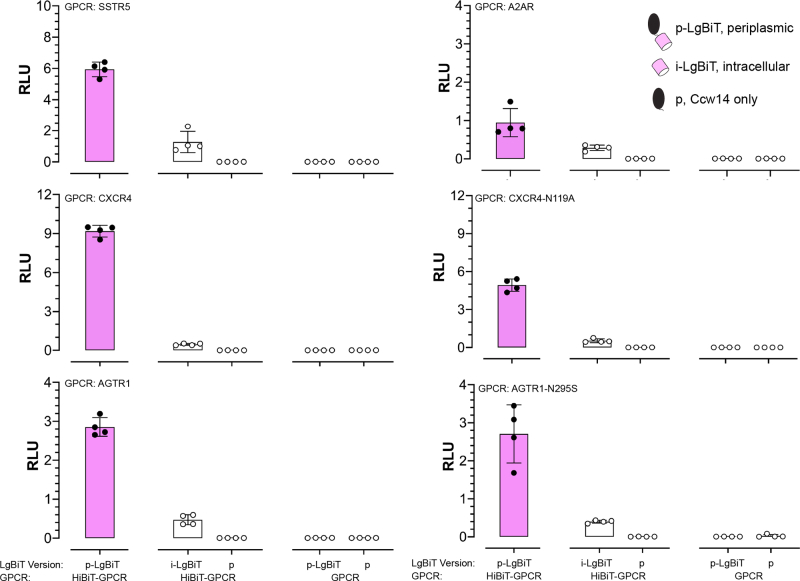


As shown in Figures 2*B* and S5, we observed robust luminescence for our control receptor, a HiBiT-tagged version of the yeast GPCR Ste2. Additional controls, including intracellular LgBiT (i-LgBiT) paired with HiBiT-Ste2, periplasmically displayed LgBiT (p-LgBiT) paired with untagged Ste2, and periplasmically displayed Ccw14 alone (p), produced no detectable signal. These results confirmed that luciferase activity is reconstituted only in the full PIONEER configuration when HiBiT binds LgBiT in the periplasmic-like space. Furthermore, these findings establish that LgBiT is a compatible Ccw14 fusion partner.

As shown in Figure 2*C*, we evaluated our surface detection method using a panel of human GPCRs that included strong (SSTR5, A2AR, and point mutants CXCR4-N119A and AGTR1-N295S) and weak (WT CXCR4 and AGTR1) DCyFIR signalers (21, 23, 24). This approach allowed us to directly test whether surface expression correlated with signaling output. p-LgBiT complemented with all receptors and produced measurable luminescence relative to i-LgBiT controls. CXCR4 produced the highest luminescence despite its weak signaling activity, whereas A2AR produced the lowest despite its strong signaling profile (23, 24). These results demonstrate the broad applicability of the assay and reveal that apparent surface expression does not necessarily predict GPCR signaling strength.

## PIONEERed agonists autocrine activate human GPCRs

**Figure 3:**
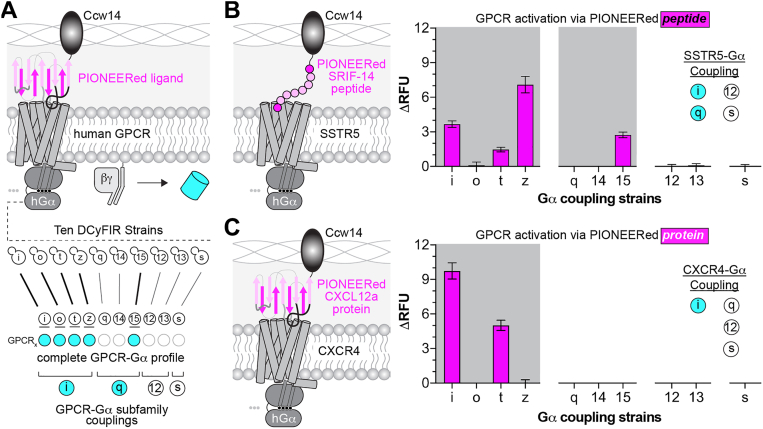
**Autocrine activation of GPCRs using the PIONEER platform.***A,* schematic of autocrine GPCR activation and downstream G protein profiling using the DCyFIR system. Ligand display *via* PIONEER enables GPCR-G_α_ coupling to be assessed by transcriptional reporter output. *B,* schematic (*left*) and functional profiling (*right*) of autocrine GPCR activation using the PIONEERed peptide ligand, SRF-14. *C,* schematic (*left*) and functional profiling (*right*) using the PIONEERed chemokine ligand, CXCL12a. GPCR activation was measured by mTq2 fluorescence and is shown as ΔRFU relative to the same strain expressing the GPCR and secretion signal only (no encoded ligand). Data represent mean ± s.d. of *n* = 4 biological replicates. PIONEER, periplasmic display of genetically encoded regulators; GPCR, G protein-coupled receptor.

For our second application, we revisited autocrine agonism of CXCR4 and SSTR5 using the final PIONEER configuration, which combines the αPrePro-ligand-Ccw14 display construct with the DCyFIR system. As shown in Figure 3*A*, we employed a panel of ten DCyFIR strains designed to test all receptor-G_α_ coupling combinations. Each strain expresses a human-yeast G_α_ chimera, created by replacing the last five residues of the yeast G_α_ C-terminus with those from a human G_α_ subunit. These chimeras span the G_i/o_ (i, o, t, z), G_q_ (q, 14, 15), G_s_ (s), and G_12/13_ (12, 13) families, enabling human GPCRs to couple efficiently to the yeast signaling pathway. As such, the DCyFIR system supports sustained activation, comprehensive pharmacological studies, and ligand/drug screening, and it incorporates an mTq2 transcriptional reporter as a fluorescent readout.

As shown in Figure 3, *B* and *C*, SSTR5 signaled through multiple G_i/o_ family members, including G_i_, G_t_, and G_z_, with G_z_ producing the strongest response. It also coupled to the G_q_ family member G_15_. CXCR4 signaled through G_i_ and G_t_, with G_i_ yielding the highest activation. These results demonstrate that the platform is robust, selective, and compatible with both peptide and protein agonists. A key advantage is that genetically encoded ligands can be produced directly in yeast through plasmid transformation or CRISPR integration, eliminating the need for external synthesis or purification. Because the ligand is retained in the periplasm, each receptor is paired with a single ligand, making the system well-suited for high-throughput library screening.

## PIONEERed nanobodies modulate human GPCR signaling

Next, we tested the compatibility of nanobody regulators with the PIONEER system. Nanobodies are single-domain antibody fragments derived from camelid heavy-chain-only antibodies that retain full antigen-binding capacity in a compact and stable format. Their small size and robustness enable efficient production in microbial systems, such as yeast, and access to epitopes often inaccessible to conventional antibodies. They are widely used in GPCR studies to stabilize specific receptor conformations, facilitating structural determination (16). They also serve as conformational biosensors, enabling the detection and modulation of GPCR activation states in live cells (35).

**Figure 4:**
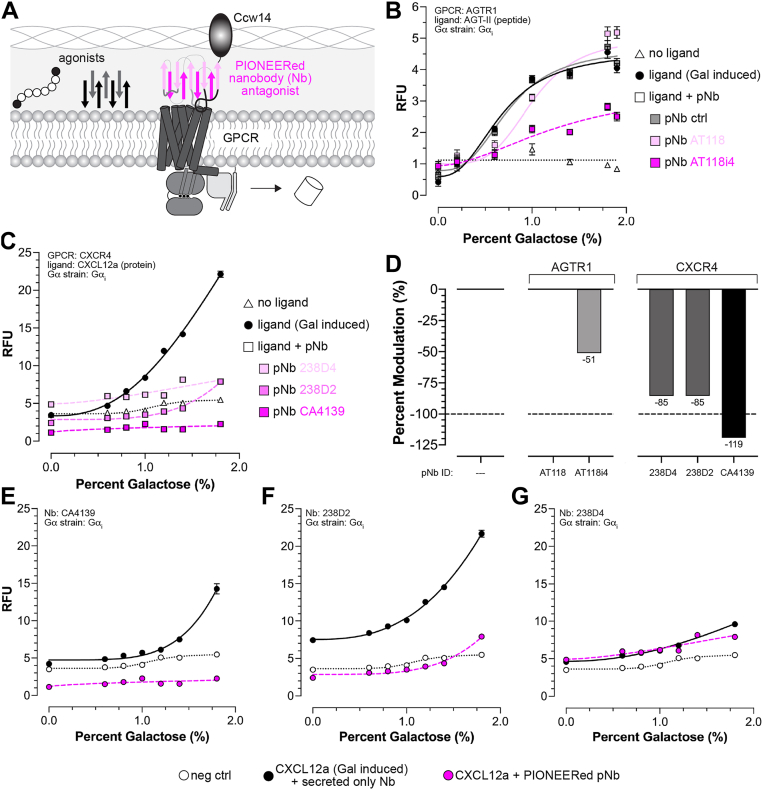
**Modulation of GPCR activity using PIONEERed nanobodies.***A,* schematic illustrating periplasmic nanobody display *via* PIONEER for antagonizing GPCRs that recognize peptide or protein ligands. *B,* inhibition of AGTR1 signaling by nanobodies displayed using PIONEER. *C,* inhibition of CXCR4 signaling by PIONEERed nanobodies. GPCRs were activated by secretion of αPrePro-tagged ligands under galactose induction. *D*, Quantification of nanobody-mediated inhibition of AGTR1 and CXCR4 from *panelsB* and *C*. For example comparison of CXCR4 inhibition by nanobodies with (*magenta*) and without (*black*) PIONEER configurations, including CA4139 (*E*), 238D2 (*F*), and 238D4 (*G*). Cells expressing CXCR4 without nanobody constructs served as a negative control. In *panelsB* and *C*, nanobody BV025 was used as a periplasmic nanobody control. Data represent mean ± s.d. of *n* = 4 biological replicates. GPCR, G protein-coupled receptor; PIONEER, periplasmic display of genetically encoded regulators; AGTR1, angiotensin receptor 1.

We reasoned that the PIONEER system would enable functional characterization of nanobody binding to GPCRs, rather than limiting analysis to binding alone. Conventional yeast surface display presents nanobodies on the outside of the cell wall and relies on purified, bead-bound GPCRs, allowing nanobodies to bind either face of the receptor without functional specificity and providing no signaling readout. In contrast, PIONEER expresses and displays nanobodies in the periplasm, linking nanobody engagement directly to GPCR signaling (Fig. 4*A*). This configuration enables selective targeting of nanobodies to either the extracellular face (extrabodies) or intracellular face (intrabodies) of the receptor, offering spatial control and functional resolution.

**Figure S6:**
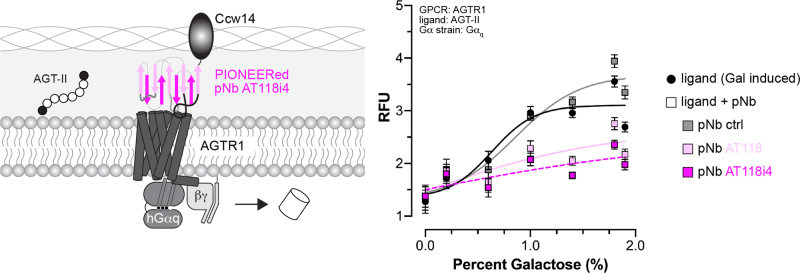


As shown in Figures 4, *B*–*D* and S6, PIONEERed extrabodies effectively inhibited AGTR1 and CXCR4 activation. To set up the assay, we placed αPrePro-AGT-II and αPrePro-CXCL12a constructs under a galactose promoter to drive inducible agonist production. By titrating galactose, we generated dose-response curves, modulating the amount of agonist released into the periplasmic space. Antagonist PIONEERed extrabodies were constitutively expressed and localized simultaneously with AGT-II and CXCL12a induction, ensuring receptors were exposed to both agonists and inhibitors. Because ligand release and extrabody engagement occurred in parallel, the assay inherently measured competition for receptor binding, enabling direct quantification of functional inhibition mediated by the PIONEERed nanobodies.

As shown in Figure 4*B*, we tested two previously developed extrabodies targeting AGTR1. The first, AT118, is a parent nanobody that binds AGTR1 but does not antagonize its activity, while the second, AT118i4, is a derivative containing two mutations in the CDR1 region that enable competitive antagonism *in vitro* and in an *in vivo* mouse model (36). Consistent with prior reports, AT118 did not inhibit AGTR1 signaling, although we observed a slight rightward shift in the dose-response curve, suggesting weak inhibition or negative allosteric modulation. As anticipated, AT118i4 robustly inhibited AGTR1 signaling, although it did not completely block receptor activation at higher agonist levels.

As shown in Figure 4*C*, we next extended our studies to three extrabodies previously developed for CXCR4. Two of these, 238D2 and 238D4 (37), competitively inhibited CXCR4-mediated signaling and antagonized the chemoattractant activity of CXCL12a. The third, CA4139 (38), was previously reported to enhance CXCR4 expression in *Pichia pastoris*, presumably by acting as a pharmacological chaperone, and in our assay competitively inhibited CXCL12a binding and reduced basal CXCR4 signaling, indicating that it functions as both an inverse agonist and competitive inhibitor. In contrast to AGTR1 inhibition by AT118i4, all three CXCR4 extrabodies maintained inhibition even at higher agonist levels. Together (Fig. 4*D*), these findings demonstrate the utility of the PIONEER platform for functionally screening and assessing GPCR antagonism by nanobodies.

To complete our analysis of PIONEERed extrabodies, we next tested whether periplasmic display was necessary. As shown in Figure 4, *E*–*G*, we examined the three CXCR4 extrabodies (CA4139, 238D2, 238D4) with and without their PIONEER configurations. CA4139 and 238D2 required display to inhibit signaling, whereas 238D4 did not. This suggested that 238D4 might bind stronger than CA4139 and 238D2. However, previous studies reported similar 238D2 and 238D4 EC_50_ values for CXCL12 ligand displacement, CXCR4-G_αqi5_-stimulated inositol phosphate accumulation, and CXCR4-G_αi_-mediated inhibition of cAMP reporter activity (37). Possible explanations include a slower off-rate for 238D4 or its higher production and secretion in the PIONEER system. Regardless, these findings demonstrate that PIONEER is necessary for capturing nanobody-GPCR interactions across a broad range of kinetic and thermodynamic properties relevant to developing pharmacological tools, biosensors, and preclinical leads.

## Intrabodies modulate GPCR signaling

**Figure 5:**
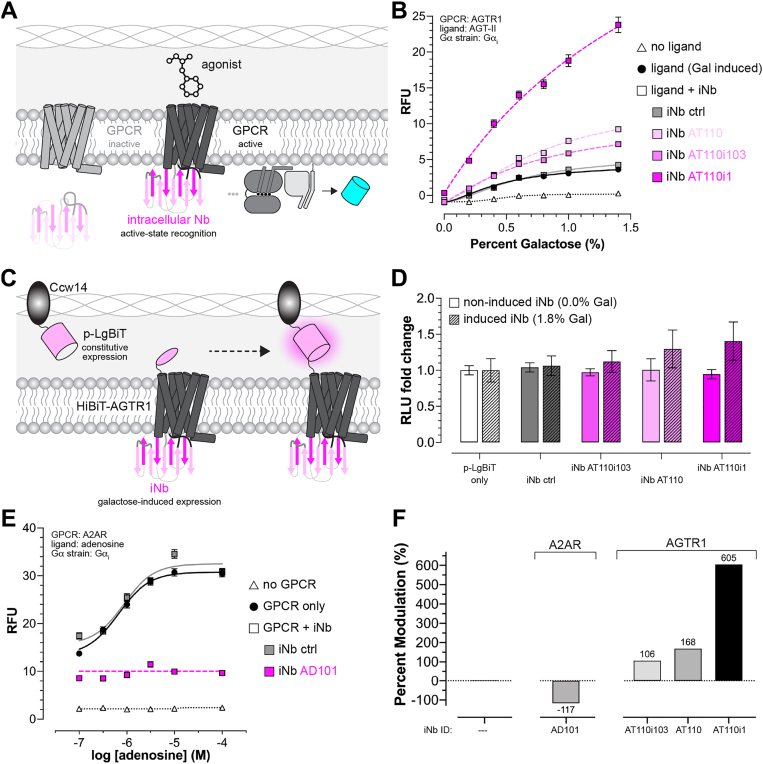
**Intracellular nanobody-mediated regulation of GPCR signaling.***A,* schematic of the platform adaptation to include intracellular nanobody (iNb) expression for modulating GPCR activity. *B,* intrabodies that enhance AGTR1 signaling upon activation by a secreted αPrePro-AGT-II ligand. *C,* schematic of PIONEER-based surface detection for monitoring iNb effects on AGTR1 surface expression. *D,* changes in AGTR1 surface expression upon induction of iNbs used in *B*. *E,* intrabodies that inhibit A2AR signaling in response to exogenous adenosine. *F,* Quantification of nanobody-mediated modulation of AGTR1 and A2AR signaling from *panelsB* and *E*. In *panelsB*, *D*, and *E*, nanobody BV025 was used as an iNb control. Data represent mean ± s.d. of *n* = 4 biological replicates. GPCR, G protein-coupled receptor; PIONEER, periplasmic display of genetically encoded regulators; AGTR1, angiotensin receptor 1.

For our final application, we leveraged PIONEER’s ability to produce nanobodies that can be secreted and displayed as extrabodies or retained inside the cell as intrabodies, which offer several advantages for GPCR studies. Intrabodies can stabilize receptors in specific active or inactive states for high-resolution structural analysis, trap transient signaling intermediates such as GPCR-G protein or GPCR-arrestin complexes, and serve as live-cell biosensors to monitor real-time conformational changes and receptor activity (35, 39, 40). To explore these capabilities, we evaluated previously reported intrabodies lacking PIONEER configurations (Fig. 5*A*), first focusing on AGTR1, for which we had already extensively characterized extrabody counterparts (Fig. 4*B*).

As shown in Figures 5*A* and S7, we evaluated three previously described AGTR1 intrabodies. The parental nanobody, AT110, and its enhanced variant, AT110i1, were developed for structural studies to bind the intracellular pocket of AGTR1 and stabilize its active conformation (41). A third intrabody from a separate study, AT110i103, was developed using autonomous hypermutation yeast surface display (AHEAD) and exhibited potent nanomolar allosteric modulation (13). In all cases, intrabody binding was characterized using radioligand allosteric shift assays, but their effects on AGTR1 signaling remained unclear.

To address this, we applied the same approach as in our extrabody studies of AGTR1. We generated dose-response curves by titrating galactose to induce AGT-II agonist expression and its secretion into the periplasmic space, with intrabodies constitutively expressed and co-localized during induction. Based on their binding to the active receptor conformation, we expected the intrabodies would inhibit signaling by competing for AGTR1’s G_α_ binding site and preventing G protein engagement. However, all three intrabodies unexpectedly enhanced pathway output in response to AGT-II.

As shown in Figure 5*B*, intrabodies AT110 and AT110i103 modestly enhanced AGTR1 signaling, approximately doubling output at peak AGT-II levels, while AT110i1 had a much stronger effect, increasing signaling more than tenfold. None of the intrabodies increased basal signaling, indicating they do not disrupt the G protein heterotrimer to release G_β_. Instead, they likely function as pharmacological chaperones, similar to the CXCR4 extrabody CA4139, by increasing the amount of receptor available for activation. If so, stronger chaperoning should correlate with greater signaling output, supporting the use of this platform to efficiently identify active-state stabilizing intrabodies for various GPCRs.

To test this, we used our PIONEER-based surface detection system to monitor changes in surface AGTR1 when iNb expression was induced (Fig. 5*C*). As expected, we observed an increase in AGTR1 surface levels when AT110, AT110i103, or AT110i1 were expressed (Fig. 5*D*), whereas no changes were detected with our control iNb. Furthermore, the relative changes in AGTR1 levels correlated with our signaling analyses from Figure 5*B*, with AT110i1 invoking the greatest effects. Together, these data supported our chaperoning hypothesis and emphasize the utility of this platform for identifying GPCR-stabilizing intrabodies.

Lastly, as shown by the adenosine dose-response curves in Figures 5*E* and S7*A*, we tested an A2AR intrabody, AD101. Like the AGTR1-directed nanobodies, AD101 recognizes the agonist-bound active state (16). However, unlike its AGTR1 counterparts, AD101 acted both as an inverse agonist and completely inhibited signaling, likely by blocking G protein recruitment. This suggests that AD101 does not increase receptor abundance by chaperoning. These results demonstrate that the platform can distinguish active-state intrabodies based on both their functional effects and chaperone ability. Intrabodies that do not alter receptor abundance may be better suited for *in vivo* biosensing, while chaperoning intrabodies could enhance dynamic range in engineered strains or support identification of high-affinity binders for structural studies. A summary of both intrabody-receptor results is shown in Figure 5*F*.

## Discussion

### Advancing periplasmic display for synthetic biology

The results presented here establish PIONEER as a versatile platform for functional screening of genetically encoded ligands and proteins using yeast periplasmic display, a conceptually rich but underexplored tool in synthetic biology. Unlike yeast surface display, which presents proteins on the outer cell wall for endpoint assays, periplasmic display localizes them to a confined, membrane-proximal space suited for functional studies. This approach is especially useful for peptides and proteins that are difficult or expensive to produce in heterologous systems and offers a scalable platform for studying targeted and naïve libraries of protein-protein interactions across diverse biotechnological applications.

Importantly, the effectiveness of PIONEERed constructs does not depend on their ability to span the periplasmic space. Ccw14-mediated cell wall binding is dynamic, with on/off kinetics that allows ligand diffusion within the compartment while preventing cargo escape. We speculate that signaling is primarily mediated by freely diffusing constructs rather than those statically tethered to the cell wall, enabling more flexible and repeated engagement with membrane-embedded targets. Building on these insights, we are exploring periplasmic display scaffolds compatible with ligands that engage their targets *via* C-terminal interactions to further expand the functional utility of the PIONEER platform.

### GPCR-specific advances and future applications

Our results also demonstrate several advances for GPCR research. Firstly, we show that PIONEERed agonists can activate human GPCRs with defined G_α_-coupling preferences, enabling comprehensive pharmacological profiling. Secondly, we demonstrate that intrabodies and PIONEERed extrabodies provide spatial precision for modulating GPCR activity through stabilization, inhibition, and chaperoning. Thirdly, we establish that the system can distinguish functional classes of nanobodies, including inverse agonists, competitive antagonists, and active-state stabilizers. These capabilities make PIONEER broadly applicable to drug discovery, biosensor development, and structural biology, where identifying conformationally selective ligands remains a significant challenge. The ability to assess peptide and protein ligand activity through signaling, rather than binding alone, marks a key advance toward function-first screening.

While the present study establishes feasibility and core functionality, we acknowledge that the potential for large-scale, fluorescence-based discovery has not yet been explicitly demonstrated. Nonetheless, our prior work with the DCyFIR platform, on which PIONEER builds, has shown that fluorescence-activated cell sorting enables robust, specific, and quantitative enrichment of functional variants in pooled library formats. These precedents support the broader scalability of PIONEER for high-throughput screening, while underscoring that the current work focuses primarily on validating the foundational framework.

### Combining AI and PIONEER

AI models are increasingly capable of generating peptides, nanobodies, and protein variants predicted to modulate biological function, yet most of these predictions remain untested in living systems. Without experimental grounding, even high-confidence models risk producing sequences that are biophysically plausible but biologically inert. PIONEER addresses this challenge by providing a genetically encoded, scalable platform that links designed sequences to functional output in a cellular context. By enabling the direct testing of AI-generated molecules for activity, inhibition, or stabilization, PIONEER delivers the functional resolution needed to evaluate and refine computational designs.

Although this study focuses on GPCRs, the PIONEER framework is agnostic to target class and broadly applicable to diverse membrane proteins, interaction partners, and signaling pathways. For example, a variety of mammalian receptor tyrosine kinases retain function when expressed in yeast (42) and have been studied using the first-generation Flo42-based autocrine design (43). As such, PIONEER can be readily adapted to these systems with minimal modification or integrated with GPCR-centric platforms such as DCyFIR to investigate GPCR–receptor tyrosine kinase crosstalk.

More generally, PIONEER provides a conceptual and experimental foundation for studying interactions between any genetically encodable proteins. While large-scale implementation will require additional optimization and benchmarking, the framework is compatible with high-throughput readouts such as fluorescence-activated cell sorting and other pooled selection strategies. Two examples include using the platform to explore and optimize split reporter systems or to design new enzyme–substrate pairs. In each case, productive complementation or enzymatic activity could, in principle, be resolved in high-throughput formats, building on the robust sorting and selection strategies established in our previous work. As AI-driven design continues to expand into more complex biological systems, frameworks like PIONEER that assess molecular function rather than only binding or structure will be essential.

## Experimental procedures

### Reagents

All media, buffers, and solutions used throughout this work are described in Table S1.

### Statistical analysis

Data are reported as mean ± sd, and all statistical analyses were performed using GraphPad Prism (https://www.graphpad.com). Comparisons between two independent groups were evaluated using an unpaired two-tailed Student’s *t* test. Comparisons involving more than two groups were assessed using one-way ANOVA. Full statistical outcomes are provided in Table S4. Statistical significance was defined as *p* < 0.05 (∗), *p* < 0.01 (∗∗), *p* < 0.001 (∗∗∗), and *p* < 0.0001 (∗∗∗∗); values with *p* > 0.05 were considered not significant (ns).

### Cell culture

NEB 5-alpha Competent *Escherichia coli*, a derivative of the DH5α strain, was used for all cloning experiments and general plasmid propagation.

All yeast strains used throughout this study are derivatives of BY4741 (MATa *his3Δ1 leu2Δ0 met15Δ0 ura3Δ0*) and are listed in Table S2. Specifically, the DCyFIR DI3Δfig1Δ::mTq2 P1 X-2 model strain (BY4741 *far1Δ0 sst2Δ0 ste2Δ0*fig1*Δ0::mTq2 X-2:P*_*TEF1a*_*-UnTS-T*_*CYC1b*_) or derivatives were used and have been described previously (24). Prior to transformation or other experimentation, yeast cells were grown at 30 °C on yeast extract peptone dextrose agar medium. Select colonies were propagated at 30 °C, static or shaking at 200 rpm, in liquid cultures of yeast extract peptone dextrose or pH-adjusted synthetic complete dextrose (SCD) low fluorescence medium (Screening Medium; pH 7.0) as specified. Transformations were performed using a standard lithium acetate protocol described previously (21). Transformants were initially selected for at 30 °C for 3 days on SCD dropout agar medium. Select transformants were then propagated at 30 °C, static or shaking at 200 rpm, in liquid cultures of SCD dropout medium. For all absorbance (*A*_600_) normalization steps, a Biomek NX^P^ liquid-handling robot was used.

### Display protein informatics

Sequences for native yeast display proteins (see Fig. S2*A*) were obtained from the *S. cerevisiae* strain S288C, the parental BY4741 strain, reference genome sequence R64-2-1 *via* the *Saccharomyces* Genome Database (44). Protein sequences were analyzed using the SignalIP 5.0 (45) and NetGPI 1.1 (46) software to identify native ss’s and GPI anchor features present, respectively. An engineered construct with a known ss and GPI anchor (Signal-NbBV025-649stalk) (16), as well as the native Gγ protein Ste18, a well-studied membrane-associated protein with no secretion sequence or GPI, were used for initial software validation.

### Cloning and molecular biology

All plasmids used and constructed throughout this study are listed in Table S3 and were validated *via* Sanger sequencing (Eurofins Genomics). All GPCR constructs were generated in a pYEplac195 vector using NEBuilder HiFi DNA Assembly Master Mix (New England Biolabs). Genes were sourced from the BY4741 genome (STE2) or the PRESTO-TANGO library (WT human GPCRs) (47). CXCR4-N119A and AGTR1-N295S mutants were first created in the PRESTO-TANGO vector *via* site-directed mutagenesis then cloned into pYEplac195 *via* HiFi DNA Assembly. These constructs were used for all GPCR assays except for Figures 2*C* and S5, as noted, as no signaling was detectable with WT versions.

Plasmids used for initial testing of display proteins were constructed by amplifying the respective genes from the BY4741 genome, starting immediately downstream of the ss cleavage site (see Fig. S2*A*). The one exception is the Flo42 construct, which contained only the C-terminal 42 amino acids of Flo1 and was previously synthesized as a gBlock by integrated DNA technologies (21). Amplicons were cloned into pYEplac181 αPre *via* HiFi DNA Assembly as αPre-*X*, where *X* is the display candidate. A yeast codon optimized mTurquoise2 (Addgene #86424), mTq2 (24, 48), was then inserted between αPre and the display protein gene in similar fashion for mTq2-based analyses (see Figs. 1, *C* and *D* and S2, *B* and *C*). For initial GPCR autocrine activation (Figs. 1, *D* and *E* and S3), SRIF-14 and CXC12a were cloned into pYEplac181 αPre-*X*, where *X* is the display candidate, *via* inverse PCR (25) or HiFi DNA Assembly, respectively, as αPre-*Y*-*X*, where *Y* is the genetically encodable ligand. The CXCL12a gene was codon optimized for *S. cerevisiae* (Integrated DNA Technologies) and sourced as a gBlock.

For mTq2-based ss testing, six unique signals were selected for comparison with aPre (see Figs. 1*F*, S4*A* and Table S3). Complete ss’s were first generated *via* Assembly PCR using 2 to 4 overlapping oligonucleotides 40 to 100 bp long. Once assembled, amplicons were directly cloned into pYEplac181 as described above, followed by cloning of mTq2 downstream of the ss . For ligand-based ss testing (see Figs. 1*F* and S4, *D*–*G*), pYEplac181 *ss*-*Y* constructs were generated *via* inverse PCR (α-factor, SRIF-14, AGT-II, CART (42-89)_(9-28), and TRV056) or HiFi DNA Assembly (CXCL12a and C5a), where *Y* is the genetically encodable ligand. The C5a gene was codon optimized for *S. cerevisiae* and sourced as a gBlock.

For PIONEER-based detection of surface GPCRs, pYEplac195 *GPCR* were N-terminally tagged with the HiBiT peptide followed by a GGSG linker *via* inverse PCR. Constructs for expressing cytosolic or PIONEERed LgBiT were created by cloning LgBiT, sourced from pBiT1.1C-LgBiT (gift from Nevin Lambert), into pYEplac181 or pYEplac181 αPrePro-Ccw14 as αPrePro-LgBiT-Ccw14 *via* HiFi DNA Assembly.

Co-expression of secreted ligands and nanobodies required designing a vector with two expression cassettes; one for constitutive expression (PIONEERed or intracellular nanobody) and one inducible (secreted ligand). Starting with pYEplac181 (constitutive *TEF1* promoter and *CYC1* terminator), an additional cassette containing the inducible *GAL1* promoter and *ADH1* terminator were inserted upstream of *TEF1* in reverse orientation *via* HiFi DNA Assembly, similar to a construct described previously (49). We refer to this construct as pYEplac181M2. *GAL1* and *ADH1* sequences were sourced from pYDS649 ^16^. Secreted ligands and nanobodies (PIONEERed, αPrePro-*nanobody*-Ccw14; intracellular, *nanobody*) were then cloned into the *P*_*GAL1*_-*T*_*ADH1*_ and *P*_*TEF1*_-*T*_*CYC1*_ expression cassettes, respectively, *via* HiFi DNA Assembly. For monitoring intracellular nanobody effects on GPCR surface expression (Fig. 5, *C* and *D*), nanobodies and αPrePro-LgBiT-Ccw14 were cloned into the *P*_*GAL1*_*-T*_*ADH1*_ and *P*_*TEF1*_*-T*_*CYC1*_ expression cassettes, respectively. In instances where GPCR activation was achieved *via* exogenous ligand application (A2AR with adenosine; see Figures 5, *E* and *F*, and S7*A*), nanobodies were expressed from the parental pYEplac181 vector. All nanobody sequences were codon optimized for *S. cerevisiae* and sourced as gBlocks.

### Data acquisition

A CLARIOstar multimode microplate reader (BMG LabTech) was used for collecting all microplate-based absorbance at 600 nm (*A*_600_), temperature, relative fluorescence units (RFU), and relative luminescence units (RLU) data with the following instrument settings: *A*_600_ (excitation 600 nm; 22 flashes/well), mTq2-based fluorescence (excitation 430/10 nm, dichroic LP 458 nm, emission 482/16 nm; 10 flashes/well; bottom read), NanoLuc luminescence (emission 482/16 nm; top read).

### Yeast growth/fitness assay

Yeast cells transformed with pYEplac181 αPre-mTq2-*X*, where *X* is the display candidate, were picked from SCD-LEU agar medium into 500 uL SCD-LEU medium in sterile 96-well deep-well blocks (Greiner Bio-One), covered with porous film (Diversified Biotech), shaken on a MixMate microplate shaker (1200 rpm for 30 s) (Eppendorf), and placed at 30 °C. After 18 h, cultures were used to prepare new 500-uL cultures normalized to *A*_600_ 0.2 and allowed to grow at 30 °C for 3 h. Finally, 50-uL cultures normalized to *A*_600_ 0.1 were prepared in a black 384-well clear-bottom plate (Greiner Bio-One) and placed in a microplate reader programmed for 30 °C incubation. Automated temperature and *A*_600_ data were collected every h for 15 h. Data were plotted in GraphPad Prism as time *versus A*_600_ and fit using a standard logistic growth equation.

### Confocal microscopy

In preparation, one yeast colony was picked from SCD dropout agar medium into 5 ml SCD dropout medium and grown overnight at 30 °C shaking (200 rpm). Cells were then washed with 5 ml SCD Screening Medium and resuspended in 400 uL, with cell pelleting (3000 g for 3 min) and harvesting prefacing each wash/resuspension step. 2 uL cell suspensions was then added to a 75 × 25 × 1-mm microscope slide and covered with a 22 × 22-mm glass coverslip (no. 1.5). Cells were imaged using an LSM800 confocal microscope (Zeiss) at 63× magnification using excitation lasers of 405 nm and processed using Zeiss’s Zen software.

### Display and secretion output and efficiency assays

For determining display protein output and efficiency, yeast strains (DI3Δfig1Δ::mTq2 P1 X-2) transformed with pYEplac181 αPre-mTq2-*X*, where *X* is the display candidate, were picked from SCD-LEU agar medium into 500 uL SCD-LEU medium in sterile 96-well deep-well blocks, covered with porous film, shaken (1200 rpm for 30 s), and grown at 30 °C. After 16 h, cultures were used to prepare new 250-uL cultures in sterile clear 96-well plates (CytoOne) normalized to *A*_600_ 0.1 in SCD Screening Medium. Plates were covered with porous film and grown at 30 °C for 21 h. After this time, 50 uL resuspended cells were transferred to a black 384-well clear-bottom plate. The remaining cultures were centrifuged (3000 g for 5 min) and 50 uL supernatant transferred to empty wells of the same 384-well plate. This plate was then centrifuged briefly, shaken (2000 rpm for 30 s), and *A*_600_ and fluorescence (gain 850) were measured to determine the degree of mTq2 secreted (supernatant) relative to total mTq2 output (cell suspension). Display efficiency was calculated using Equation 1, where F_s_ is the relative fluorescence of the supernatant for αPre-mTq2-*X* (F_sX_) or empty vector (F_s0_), and F_t_ is the total relative fluorescence of αPre-mTq2-*X* (F_tX_) or empty vector (F_t0_), where *X* is the display candidate.

(1) $$\%\;\text{Display}\;\text{Efficiency}=100\;-\;\left(\left(\frac{F_{\text{sX}}\;-\;F_{s0}}{F_{\text{tX}\;}-{\;F}_{t0}}\right)100\right)$$

For determining output and efficiency of ss’s, pYEplac181 *ss*-mTq2 constructs were transformed, cells prepared, experiments performed, and data collected as described above. Total fluorescence of the cell suspensions was reported as total protein output, whereas secretion efficiency was calculated using Equation 2. F_sX_ and F_tX_ are the relative fluorescence of the supernatant or total cell suspension of pYEplac181 *ss*-mTq2 transformants, respectively.

(2) $$\%\;\text{Secretion}\;\text{Efficiency}=\left(\frac{F_{\text{sX}}\;-\;F_{s0}}{F_{\text{tX}\;}-{\;F}_{t0}}\right)100$$

### PIONEER-based split luciferase assay for surface receptor detection

Yeast cells co-transformed with pYEplac181 αPrePro-LgBiT-Ccw14 (p-LgBiT) and pYEplac195 HiBiT-*X*, where *X* is the GPCR, were picked from SCD-URA-LEU agar medium into 1 ml SCD-URA-LEU medium in sterile 96-well deep-well blocks, covered with porous film, shaken (1200 rpm for 30 s), and placed at 30 °C. Cotransformations of HiBiT-GPCRs with pYEplac181 i-LgBiT or PIONEER only (pYEplac181 p) and non-tagged GPCRs (pYEplac195 *GPCR*) with pYEplac181 p or p-LgBiT were included as controls (see Figs. 2, *B* and *C* and S5). After 21 h, cultures were used to prepare new 200-uL cultures normalized to *A*_600_ 0.2 in SCD Screening Medium in sterile clear 96-well plates, covered with porous film, and placed at 30 °C. After 15 h, cultures were used to prepare 80-uL cultures normalized to *A*_600_ 1.0 in a black, black-bottom 384-well plate (Greiner Bio-One) with furimazine (1:1000). Plate was shaken (2000 rpm for 30 s) and luminescence measurements (gain 3000) were performed in a microplate reader. Data were plotted as relative luminescence units (RLU) and analyzed in GraphPad Prism.

For monitoring changes in AGTR1 surface levels in the presence of intracellular nanobodies (Fig. 5, *C* and *D*), yeast cells co-transformed with pYEplac195 HiBiT-AGTR1 and a pYEplac181M2-derived construct dually expressing αPrePro-LgBiT-Ccw14 (*P*_*TEF1*_-αPrePro-LgBiT-Ccw14*-T*_*CYC1*_) and a nanobody under galactose regulation (*P*_*GAL1*_-*nanobody-T*_*ADH1*_) were prepared in SCD-URA-LEU medium in similar fashion. However, after the initial 21-h growth, cultures were used to prepare new 1.5-mL cultures in low-glucose SCD-URA-LEU medium (1.9% galactose, 0.1% glucose) in sterile 96-well deep-well blocks normalized to *A*_600_ 0.2. Blocks were covered with porous film, shaken, and placed at 30 °C. After 21 h, low-glucose cultures were used to prepare new 200-uL cultures normalized to *A*_600_ 0.2 in sterile clear 96-well plates in either SCD Screening Medium without galactose or 1.8% galactose, maintaining a total sugar content of 2.0%. The plate was covered with porous film and placed at 30 °C for 15 h. Finally, cultures were used to prepare 80-uL cultures normalized to *A*_600_ 1.0 in a white, white-bottom 384-well plate (Greiner Bio-One) with furimazine (1:1000). Plate was shaken (2000 rpm for 30 s) and luminescence measurements (gain 3000) were performed in a microplate reader. Data were plotted as RLU and analyzed in GraphPad Prism. Fold change in luminescence was calculated relative to p-LgBiT only (no nanobody) in the matched sugar-specific medium.

### GPCR activation assay – exogenous ligand application

For achieving GPCR activation *via* exogenous ligand application, receptor expression constructs (pYEplac195 *GPCR*) were transformed into the DI3Δfig1Δ::mTq2 P1 X-2 yeast strain (pYEplac195 Ste2) or derived DCyFIR strains (Table S2). Resulting transformants were picked from SCD-URA agar medium into 1 ml SCD-URA medium in sterile 96-well deep-well blocks, covered with porous film, shaken (1200 rpm for 30 s), and placed at 30 °C.

After 18 to 21 h, cultures were used to prepare new 72-uL duplicate cultures in a sterile black 384-well clear-bottom plate normalized to *A*_600_ 0.11 in SCD Screening Medium. Following this, 8 uL of 10× drug or vehicle (H_2_O) were added per well. The plate was briefly centrifuged, shaken (2000 rpm for 30 s), and placed in a microplate reader programmed for 30 °C incubation. Automated temperature, *A*_600_, and mTq2 fluorescence (gain 1200) data were collected every h for 15 h.

Data were plotted in GraphPad Prism as fluorescence *versus A*_600_ and fit *via* linear regression to generate slope and intercept values for each treatment condition, which were used to extrapolate fluorescence to a standardized *A*_600_ of 1.0 and reported as RFU. The resulting RFU data were then plotted as a function of log [ligand] and fit using the four-parameter pharmacological function log(agonist) *versus* response. Where applicable, error bars represent the standard deviation of the fitted slopes.

### Autocrine GPCR activation assay – secreted and PIONEERed ligands

For initially achieving autocrine GPCR activation, GPCR expression constructs were co-transformed with constructs for ligand secretion (pYEplac181 *ss-ligand*) or secretion and periplasmic display (pYEplac181 *ss-ligand-display*) into the DI3Δfig1Δ::mTq2 P1 X-2 yeast strain (pYEplac195 Ste2) or derived DCyFIR strains. Resulting transformants were picked from SCD-URA-LEU agar medium into 1 ml SCD-URA-LEU medium in sterile 96-well deep-well blocks, covered with porous film, shaken (1200 rpm for 30 s), and placed at 30 °C. After 18 to 21 h, cultures were used to prepare new 80-uL cultures in a sterile black 384-well clear-bottom plate normalized to *A*_600_ 0.1 in SCD Screening Medium and placed in a microplate reader programmed for 30 °C incubation. Automated data collection and analyses were performed as described above.

### Nanobody-based modulation of GPCR signaling

For testing GPCR functional modulation by both extracellular- and intracellular-binding nanobodies, GPCR expression constructs were typically co-transformed with pYEplac181M2-derived constructs dually expressing a defined nanobody (*P*_*TEF1*_-αPrePro-*nanobody*-Ccw14*-T*_*CYC1*_ or *P*_*TEF1*_-*nanobody-T*_*CYC1*_) and a secreted ligand under galactose regulation (*P*_*GAL1*_-αPrePro-*ligand-T*_*ADH1*_) into specified DCyFIR strains. For instances where ligands were added exogenously, nanobodies were expressed from pYEplac181M2 constructs without encoded ligands (*P*_*GAL1*_*-T*_*ADH1*_). Resulting transformants were picked from SCD-URA-LEU agar medium into 1 ml SCD-URA-LEU medium in sterile 96-well deep-well blocks, covered with film and shaken, and placed at 30 °C for 18 to 21 h. Cultures were then used to prepare new 1.5-mL cultures in low-glucose SCD-URA-LEU medium (1.9% galactose, 0.1% glucose) in sterile 96-well deep-well blocks normalized to *A*_600_ 0.2. Blocks were covered with porous film, shaken, and placed at 30 °C.

After 18 to 21 h, low-glucose cultures were used to prepare new 80-uL cultures in a sterile black 384-well clear-bottom plate normalized to *A*_600_ 0.1 in one of eight SCD Screening Medium solutions with varying galactose/glucose ratios (see Table S1), maintaining a total sugar content of 2.0%. The plate was then placed in a microplate reader programmed to 30 °C incubation and automated temperature, *A*_600_, and mTq2 fluorescence (gain 1200) data were collected every h for 15 h.

Data were plotted in GraphPad Prism as fluorescence *versus A*_600_ and fit *via* linear regression to generate slope and intercept values for each treatment condition, *i.e.*, galactose/glucose ratio (for secreted ligands) or ligand concentration (exogenous ligand applications), which were used to extrapolate fluorescence to a standardized *A*_600_ of 1.0 and reported as RFU. The resulting RFU data were then plotted as either a function of galactose percentage and fit using the four-parameter pharmacological function agonist *versus* response (secreted ligands), or as a function of log[ligand] and fit using the pharmacological function log(agonist) *versus* response (exogenous ligand applications). Where applicable, error bars represent the standard deviation of the fitted slopes.

## Data availability

All of the data for this study are available in the manuscript and its supporting information.

## Supporting information

This article contains supporting information.

## Conflict of interest

D.G.I. and J.B.R. are inventors on a pending U.S. utility patent application (filed November 29, 2023) related to the PIONEER platform. The other authors declare no competing interests.

## Figures and Tables

**Figure S1:**
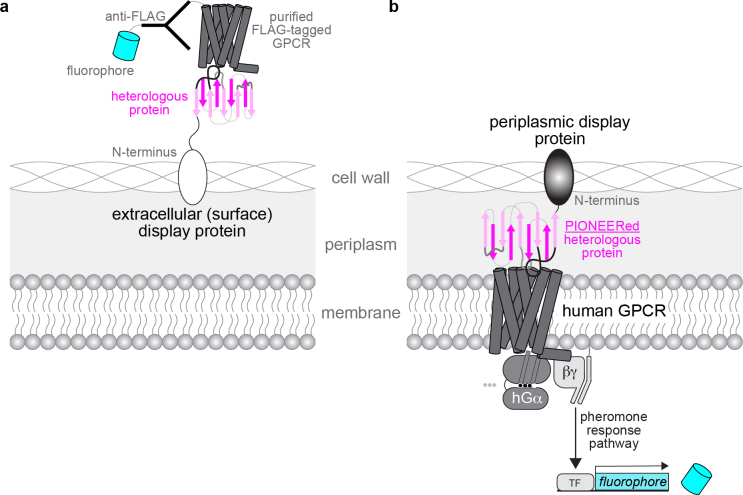


**Figure S7:**