# Supplementary material for: PIONEER: A periplasmic display platform for synthetic biology-based screening of genetically encoded protein regulators
Source: J Biol Chem. 2025 Nov 20;302(1):110967. doi: 10.1016/j.jbc.2025.110967 (PMC12796099; doi:10.1016/j.jbc.2025.110967)
Supplement: Supplementary Figure Legends [file mmc2.pdf]

Supporting Information Figure Legends

Fig. S1 | Yeast surface versus PIONEER-based protein display
a, Schematic of conventional yeast display systems, where heterologous proteins are presented on the exterior of the cell wall.
b, Schematic of the PIONEER system, which displays proteins in the periplasmic space between the plasma membrane and cell wall.
Pairing PIONEER with functional yeast signaling platforms, such as DCyFIR, enables detection of physiologically relevant protein-receptor interactions.

Fig. S2 | Characterization of periplasmic display proteins for PIONEER
a, Sequence analysis of candidate display proteins to identify native secretion signals (black line with grey shading), predicted cleavage sites (magenta line), and glycosylphosphatidylinositol (GPI) anchor motifs (black dashed line with grey mesh shading). Amino acid positions corresponding to cleavage and GPI anchor sites are indicated. Signal-NbBV025-649stalk and *Ste18* were included as positive (synthetic design with known secretion signal and GPI anchor) and negative (membrane-bound, non-secreted) controls, respectively.
b, Quantification of mTq2 fluorescence from tagged display proteins to assess expression levels. “neg ctrl” indicates cells transformed with empty vector. Data are presented as mean ± s.d. of $n = 8$ biological replicates.
c, Display efficiency calculated as the percentage of mTq2 fluorescence retained within the cell relative to total mTq2 (intracellular plus secreted). Data are presented as mean ± s.e.m. of $n = 8$ biological replicates. Data from **b** and **c** were used for protein performance scoring in Fig. 1d.

Fig. S3 | Autocrine GPCR-G_α activation profiles with displayed ligands
a, Autocrine activation of SSTR5 by secretion or combined secretion and periplasmic display of its cognate ligand fused to Flo42, Ccw14, Sed1, or Suc2.
b, Autocrine activation of CXCR4 under the same conditions.

Data represent mean ± s.d. of $n = 4$ biological replicates. Experiments were performed using DCyFIR strains and results contributed to display protein scoring shown in Fig. 1d-e.

Fig. S4 | Characterization of secretion signals for PIONEER system design
a, Amino acid sequences of secretion signals selected for evaluation.
b, Quantification of secreted mTq2 fluorescence (relative fluorescence units, RFU) to assess secretion signal output. “neg ctrl” indicates cells transformed with empty vector. Data are shown as mean ± s.d. of $n = 8$ biological replicates.
c, Secretion efficiency reported as the percentage of mTq2 detected in the medium relative to total mTq2 (intracellular plus secreted). Data are shown as mean ± s.e.m. of $n = 8$ biological replicates.
d-f, Autocrine activation of SSTR5 (**d**), AGTR1 (**e**), and CXCR4 (**f**) in response to secretion of their cognate ligands using the indicated secretion signals.
g, Additional autocrine signaling profiles driven by ligands secreted via the αPrePro signal. Fluorescence is reported as ΔRFU relative to strains expressing the GPCR and secretion signal only (no encoded ligand).
h, Estimation of ligand secretion levels produced with αPrePro. Titration curves for *Ste2* (left) and SSTR5 (right) using exogenous ligands were used to estimate concentrations of secreted αPrePro-ligands (dashed line) based on fluorescence output from Fig. 1f. SSTR5 experiments were conducted in the DCyFIR $G_{\alpha i}$ strain; *Ste2* experiments were performed in the parental strain with native G_{α} (*Gpa1*).

Data in **d-h** are shown as mean ± s.d. of $n = 4$ biological replicates. Results from **b-f** were used to score secretion signal performance in mTq2 (**b-c**) and ligand-secretion (**d-f**) assays, as presented in Fig. 1f.

Fig. S5 | Control experiments for PIONEER-based detection of surface GPCRs
Control data corresponding to Fig. 2c. Luminescence is generated by reconstitution of NanoLuc between periplasmically displayed LgBiT and HiBiT-tagged GPCRs expressed at the cell surface. Luminescence is reported in relative luminescence units (RLU).

Data represent mean ± s.d. of $n = 4$ biological replicates.

Fig. S6 | AGTR1-$G_{\alpha q}$ antagonism by PIONEERed nanobodies AT118 and AT118i4
Schematic and functional data showing inhibition of AGTR1-$G_{\alpha q}$ activation by angiotensin II (AGT-II) using PIONEERed nanobodies AT118 and AT118i4. This experiment parallels the $G_{\alpha i}$-based assays shown in Fig. 4b.

Data represent mean ± s.d. of $n = 4$ biological replicates.

Fig. S7 | GPCR modulation by intracellular nanobodies
a, Schematic and functional data showing that intracellular nanobody AD101 reduces A2AR-$G_{\alpha 15}$ signaling. This mirrors its inhibitory effect on A2AR-$G_{\alpha i}$ signaling shown in Fig. 5e.
b, Schematic and data demonstrating that nanobody AT110i1, specific for AGTR1, does not enhance CXCR4-$G_{\alpha i}$ signaling in response to CXCL12a. This contrasts with the strong potentiation of AGTR1-$G_{\alpha i}$ signaling by AT110i1 shown in Fig. 5b.

Data represent mean ± s.d. of $n = 4$ biological replicates.

Supporting Information Table Legends

Supporting Table 1. Recipes. Yeast media recipes and reagents.

Supporting Table 2. Strains. Yeast strain genotypes.

Supporting Table 3. Plasmids. Yeast plasmids.

Supporting Table 4. Stats. Details for statistical analyses.
